# Virtual Hematoxylin and Eosin Transillumination Microscopy Using Epi-Fluorescence Imaging

**DOI:** 10.1371/journal.pone.0159337

**Published:** 2016-08-08

**Authors:** Michael G. Giacomelli, Lennart Husvogt, Hilde Vardeh, Beverly E. Faulkner-Jones, Joachim Hornegger, James L. Connolly, James G. Fujimoto

**Affiliations:** 1 Department of Electrical Engineering and Computer Science and Research Laboratory of Electronics, Massachusetts Institute of Technology, Cambridge, Massachusetts, 02139, United States of America; 2 Pattern Recognition Lab, Friedrich-Alexander University Erlangen-Nürnberg (FAU), Erlangen-Nürnberg, Germany; 3 Department of Pathology, Beth Israel Deaconess Medical Center, Harvard Medical School, Boston, Massachusetts, United States of America; 4 Graduate School in Advanced Optical Technologies, Friedrich-Alexander University Erlangen-Nürnberg (FAU), Erlangen-Nürnberg, Germany; Pennsylvania State Hershey College of Medicine, UNITED STATES

## Abstract

We derive a physically realistic model for the generation of virtual transillumination, white light microscopy images using epi-fluorescence measurements from thick, unsectioned tissue. We demonstrate this technique by generating virtual transillumination H&E images of unsectioned human breast tissue from epi-fluorescence multiphoton microscopy data. The virtual transillumination algorithm is shown to enable improved contrast and color accuracy compared with previous color mapping methods. Finally, we present an open source implementation of the algorithm in OpenGL, enabling real-time GPU-based generation of virtual transillumination microscopy images using conventional fluorescence microscopy systems.

## Introduction

Classical histopathology is the gold standard for clinical evaluation of many suspected neoplasms, including cancer of the breast. In histopathology, the tissue specimen is fixed, dehydrated, paraffin embedded, sectioned into micron thin slices, and then visualized using molecularly-specific absorptive dyes in a transillumination light microscope. The most commonly used dyes, hematoxylin and eosin (H&E), stain the cell nucleus purple and supporting stroma and cytoplasm pink. Unfortunately, the complex specimen preparation requires processing times on the order of one day, which precludes using conventional histopathology for real-time applications such as surgical or biopsy guidance [1]. The lack of real-time information on pathology may necessitate a second surgery or biopsy procedure in the event that the resection or sampling proves insufficient [2–4]. Repeat procedures pose additional risk to patients, may delay adjuvant therapy, reduce cosmetic outcomes, and impose an additional financial burden on the healthcare system [5,6]. To address this problem, various groups have investigated fluorescence microscopy techniques such as confocal microscopy [7,8], multiphoton microscopy [9,10], and structured illumination microscopy [11]. These techniques have the advantage of epi-illumination and optical depth sectioning, avoiding the need for time-consuming fixation and processing steps, potentially enabling real-time assessment of pathology.

In order to facilitate clinical interpretation of fluorescence microscopy images by pathologists, multiple groups have demonstrated virtual-H&E rendering, in which fluorescence or reflectance intensity values are color mapped analogously to H&E histopathology [7,9,12–15]. Other techniques such as virtual-H&E using intrinsic contrast have also been demonstrated, suggesting possible *in vivo* applications [16]. In previous work, we have demonstrated that virtual-H&E rendering of multiphoton microscopy (MPM) images achieves 95.4% sensitivity and 93.3% specificity for assessing malignancy of the breast as compared to conventional histopathology, suggesting that virtual-H&E techniques may be a powerful method for evaluating surgical pathology [9]. However, relatively few groups have published complete algorithms for virtual-H&E rendering, and most previous algorithms have been based on additive blending, in which the hue at each pixel is displayed as the superposition of the assumed hues of each absorptive dye [12,17]. Adding the transmission spectra of dyes is not a physically realistic model of light propagation in transillumination microscopy, and yields unphysical results, such as predictions of negative color channel intensity for images that have spectrally overlapping dyes. Typically, the unphysical pixel values produced by non-physical models of absorption are addressed by clamping to zero or renormalization at the expense of reduced dynamic range and color accuracy.

To address these limitations, we demonstrate a physically realistic rendering approach based on modeling transillumination absorption using the Beer-Lambert law. In this approach, we compute the transmission T of a wavelength λ through a histology specimen slide containing N absorbing dyes:

$$T_{\lambda }=exp{\left(-\overset{N}{\underset{i=1}{\sum }}\sigma_{\lambda i}\overset{l}{\underset{0}{\int }}n_{i}\left(z\right)dz\right)}^{}$$ (1)

where *σ*_λi_ is the wavelength dependent attenuation cross section for the i*th* dye, *l* is the specimen thickness, and n_i_ is the volumetric concentration of dye. Recognizing that the quantity $\overset{l}{\underset{0}{\int }}n_{i}\left(z\right)dz$ is the concentration of dye integrated through the specimen thickness, Eq 1 becomes:

$$T_{\lambda }=exp{\left(-\overset{N}{\underset{i=1}{\sum }}\sigma_{\lambda i}c_{i}\right)}^{}$$ (2)

where *c*_*i*_ represents the thickness integrated concentration of the *i*th dye per area on the slide. Therefore, the transmission for a given wavelength is exponential in the product of the dye concentration per area and a constant representing the spectral properties of each dye. Because the emitted fluorescent intensity from a volume of tissue at realistic fluorophore concentrations is approximately linear with fluorophore concentration, an epifluorescence intensity measurement *I*_*i*_ and an arbitrary scaling constant *k* that accounts for the detector sensitivity, gain, etc. can be substituted for each dye concentration:

$$T_{\lambda }=exp{\left(-\overset{N}{\underset{i=1}{\sum }}\sigma_{\lambda i}I_{i}k\right)}^{}$$ (3)

Since the objective is to render a digital image, the optical transmission T_λ_ can be replaced with a digital pixel intensity T_Virtual_ at each of *M* color channels:

$$T_{\text{Virtual},M}=exp{\left(-\overset{N}{\underset{i=1}{\sum }}\beta_{M,i}I_{i}k\right)}^{}$$ (4)

where the wavelength dependent attenuation cross section is replaced with β_M,i_, the attenuation of the *i*th dye integrated over the spectral range of the *M*th color channel. Conceptually this step corresponds to converting from a model of transmitted light as a continuous function of wavelength to a discrete representation of *M* individual color channels, with β_M_ representing the attenuation of the *i*th dye over the range of wavelengths covered by the *M*th channel. If high quality hyperspectral data for each dye is available, hyperspectral measurements of the dye transmission could be scaled to obtain the values of β_M_ directly, and the final RGB output color space values for each pixel computed using color matching functions [15]. However, since most dyes do not have extremely sharp spectral features, it is conceptually simpler to directly compute the virtual transmission in the output color space (most commonly sRGB). For the special case of a virtual transillumination H&E microscopy image computed in a trichromatic RGB colorspace using a DNA-specific agent and eosin as fluorophores, this becomes:

$$R=exp{\left(-\beta_{hematoxylin,red}I_{DNA}k\right)}^{}exp{\left(-\beta_{eosin,red}I_{eosin}k\right)}^{}$$ (5)

$$G=exp{\left(-\beta_{hematoxylin,green}I_{DNA}k\right)}^{}exp{\left(-\beta_{eosin,green}I_{eosin}k\right)}^{}$$ (6)

$$B=exp{\left(-\beta_{hematoxylin,blue}I_{DNA}k\right)}^{}exp{\left(-\beta_{eosin,blue}I_{eosin}k\right)}^{}$$ (7)

Where the 6 *β* values in (Eqs 5–7) represent the R, G, and B color coordinates of pure hematoxylin and eosin expressed in the chosen colorspace. (Table 1, *β* values matched to the example histology specimen for sRGB). The stains and corresponding *β* values will vary slightly for specimens prepared in different pathology labs.

**Table 1:** Reference *β* values expressed in the sRGB color space

	*Eosin*	*Hematoxylin*
*Red*	*0*.*050*	*0*.*860*
*Green*	*1*.*000*	*1*.*000*
*Blue*	*0*.*544*	*0*.*300*

Although this model is physically accurate, a negative exponential approaches 0 asymptotically, whereas a computer display has a finite number of levels, typically 256, and a limited contrast ratio. Using the sRGB color space, we can truncate the exponential decay for each absorber at 0.0075 times the peak display optical intensity while setting k = 2.5. Because each color channel is the product of absorption from two dyes as well as the sRGB gamma, this is equivalent to assuming a maximum contrast ratio for a color channel of approximately 1000:1, and should not require adjustment unless an alternative color space is used. The exact value of k has only a minor effect, because shifting the absolute level of the darkest pixels only slightly changes the image appearance.

In comparison to previous methods, the virtual transillumination microscopy algorithm derived here has several important properties. First, the virtual transillumination microscopy algorithm uses a relative optical attenuation calculated from the fluorophore concentration and the simulated optical flux, rather than adding an absolute signal. Consequently, while an additive model will predict negative intensities and will have reduced dynamic range if multiple spectrally-overlapping dyes absorb strongly at the same location, the virtual transillumination algorithm can accurately model an unlimited number of absorbing species with potentially overlapping spectra without loss of contrast or unphysical results. Second, because the intensity in a transillumination microscope is nonlinear with concentration, the virtual transillumination algorithm also nonlinearly maps epifluorescence into virtual absorption, resulting in a physically realistic level of contrast. Finally, we note that since the dynamic range of most computer monitors (64–256 shades per color channel) is much lower than the dynamic range of microscopy fluorescence measurements (500–1000 or more intensity levels), the virtual transillumination microscopy algorithm intrinsically utilizes all of the available dynamic range even when displayed on a monitor with a limited number of colors, without the need for manual color adjustments, contrast enchantment, or other image processing.

## Methods

### GPU Implementation

**Fig 1:**
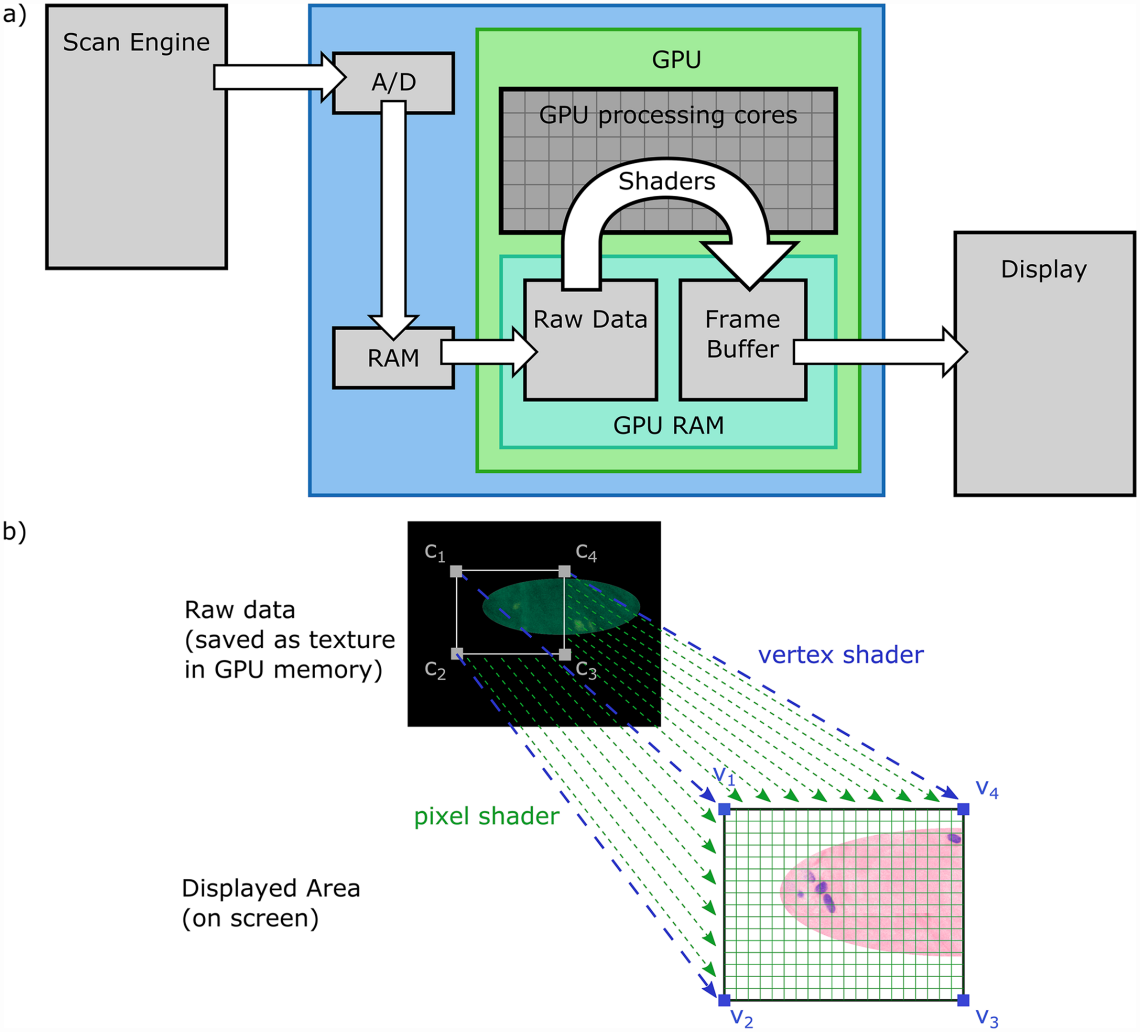
Schematic diagram of the OpenGL virtual transillumination rendering algorithm. a) Data flow in the OpenGL algorithm showing pixel data generated by the scan engine and A/D being processed by shaders. Fluorescence data is loaded into GPU RAM as a texture, processed by a shader running on hundreds or thousands of GPU cores and the final result is stored in the frame buffer for display. b) The relationship between vertex and pixel shaders. The vertex shader defines the quad’s position on screen and provides a mapping to the texture coordinates. Gray squares c_1_ to c_4_ show texture coordinate locations in GPU memory, while the associated vertices v_1_ to v_4_ are shown in blue. Blue dotted arrows show the association of the texture coordinates to the vertices by the vertex shader. The pixel shader performs the computations according to Beer’s law individually for each displayed pixel. The green grid indicates the pixel grid of the final image, while the green dotted arrows show the transform by a vertex shader which is run for each pixel.

In order to implement real-time virtual H&E display of fluorescence data, we chose a GPU-based implementation using OpenGL. GPUs possess massively parallel processing capabilities optimized for the display of complex visual data with very low latency, making them ideal for the implementation of virtual histology rendering (Fig 1(a)). Programs executed by the GPU, called shaders, are run in parallel across large datasets and offer programmability approaching that of conventional CPUs. Our implementation utilizes two OpenGL concepts, the vertex shader and pixel shader, which are shown in Fig 1(b). The vertex shader defines a surface on which a virtual transillumination microscopy image is rendered and provides features such as scaling of image data to the monitor resolution and rotation or mirroring of the image data to match the physical geometry of the microscope. The pixel shader performs the actual Beer’s law calculations for the given combination of dyes to generate a virtual transillumination H&E image which is projected onto the surface defined by the vertex shader.

Our algorithm implements the following steps using OpenGL. First, multiphoton data is loaded into GPU texture memory. A vertex shader is used to position a quad, a rectangular polygon that serves as the display surface. The quad resizes the (arbitrary) microscope resolution to the screen resolution by defining a mapping to a texture coordinate (Fig 1(b), gray squares) for every vertex of the quad (blue squares). Using the mapping defined by the vertex shader (blue dotted lines), the pixel shader interpolates texture values onto each pixel in the output frame (green dotted lines). The interpolated values are then converted into virtual H&E images by applying Beer’s law. In this example, two fluorescent channels, corresponding to eosin (itself a fluorophore) and any fluorescent hematoxylin analog are used to estimate the chromophore concentrations in tissue. Because of the parallel nature of the OpenGL implementation, thousands of pixel values can be computed in parallel, and millions of parallel groups of pixels can be sequentially processed per second. Therefore, tens of billions of pixels can be processed per second on consumer-grade GPU hardware, enabling processing delays to be reduced to sub-millisecond intervals per image frame. In contrast, if this mapping algorithm was run on a regular CPU, the calculations could only be performed for a comparatively small number of pixels in parallel (namely, the number of CPU cores), which would greatly increase the image latency and reduce the maximum number of pixels per second that could be displayed by several orders of magnitude.

A complete example implementation of the virtual transillumination algorithm in OpenGL using the reference *β* values as well as sample image data can be downloaded from https://github.com/mgiacomelli/VirtualHE.

### Sample Preparation and Imaging

All tissue was imaged under a protocol approved by the Massachusetts Institute of Technology Committee on the Use of Humans as Experimental Subjects (COUHES) and the Beth Israel Deaconess Medical Center (BIDMC) Committee on Clinical Investigations (CCI). Surgical specimens which were discarded and not required for diagnosis were de-identified prior to enrollment by non-study personnel, transported to MIT in chilled RPMI solution, and dissected to expose relevant pathology. Specimens were then labeled with DAPI (a widely used fluorescent hematoxylin analog) and eosin and then fixed in formalin to enable repeated imaging over an extended period. DAPI was chosen because it is widely used in microscopy, however many other nuclear contrast agents could be used along with the appropriate filters. Total sample preparation excluding fixation was less than 3 minutes, substantially less than the ~1 day required for conventional histopathology processing. Following fixation, specimens were selected and imaged with a commercial multiphoton microscope (Thorlabs, Inc.) with a XLUMPFL20XW 1.0 NA 20x objective (Olympus) and a titanium sapphire laser (Chameleon, Coherent, Inc.) operating at 780 nm. The microscope generated images of 1024 by 1024 pixels at 16 frames per second. The axial sectioning resolution in tissue was approximately 1 micron. Detection was performed using H7422-40P photomultiplier tubes (Hamamatsu, Inc.) with a 510 nm dichroic beam splitter and a 460/50 nm emission filter (DAPI channel) and a 590/100 nm filter (eosin). To reduce the confounding effects of detector noise on image quality between linear and nonlinear rendering methods, each frame was averaged 16 times to reduce noise. To overcome the limited field of view of conventional multiphoton microscopy, a high speed translation stage (MLS203, Thorlabs, Inc.) was used to mosaic multiple, 500 micron size fields with 50% overlap using custom acquisition software written in C++.

### Image Processing and Normalization

Following acquisition, histograms were computed of both the eosin and DAPI fluorescence signal data across all frames in the mosaic. Additional gain was then applied such that 1 additional pixel per 100,000 was saturated at maximum intensity. This additional gain, on the order of 0–20% for typical data sets, ensures that each mosaic was similarly scaled in each fluorescence channel. In analogy to H&E processing, this would be equivalent to ensuring that consistent concentrations of hematoxylin and eosin were used to process each specimen. The mosaic image data was then processed using the Beer’s law virtual transillumination algorithm, and two other methods described in the literature, an additive method with a nonlinear intensity transfer function [9], and an additive method with a linear intensity transfer function [12]. No further pre or post-processing was applied, although any predictions of absorption greater than unity by additive methods were clamped at zero transmission in order to maximize image contrast. Following generation of virtual H&E frames using each algorithm, stitching and blending of individual fields was performed using photograph stitching software (Image Composite Editor, Microsoft Research).

## Results

### Comparison to H&E Histology

To demonstrate the virtual transillumination algorithm, we present images from discarded surgical breast mastectomy specimens from a patient undergoing mastectomy for invasive ductal carcinoma. A solid tumor was identified, and then a region of apparently normal stroma several millimeters from the tumor boundary was rapidly stained, fixed and then imaged with MPM. The specimen was mounted in a specially modified histology cassette fitted with a coverslip to facilitate imaging, while a sponge soaked in phosphate-buffered saline (PBS) was used to keep the specimen hydrated. To facilitate co-registration with subsequent H&E sectioning planes, multiple Z sections were imaged at 5 micron depth intervals from approximately 10 microns depth to 40 microns, the maximum imaging depth to which the stain penetrated uniformly during the brief staining interval. Following MPM imaging, the specimens were processed and carefully embedded in paraffin with the MPM imaging plane oriented parallel to the paraffin block face. The paraffin block was cut into 5 micron sections (approximately 4–5 times the multiphoton axial section thickness) which were coregistered with the MPM images. Note that some diffuse eosin staining is present in the surrounding PBS, resulting in a faint pink hue surrounding the specimen.

**Fig 2:**
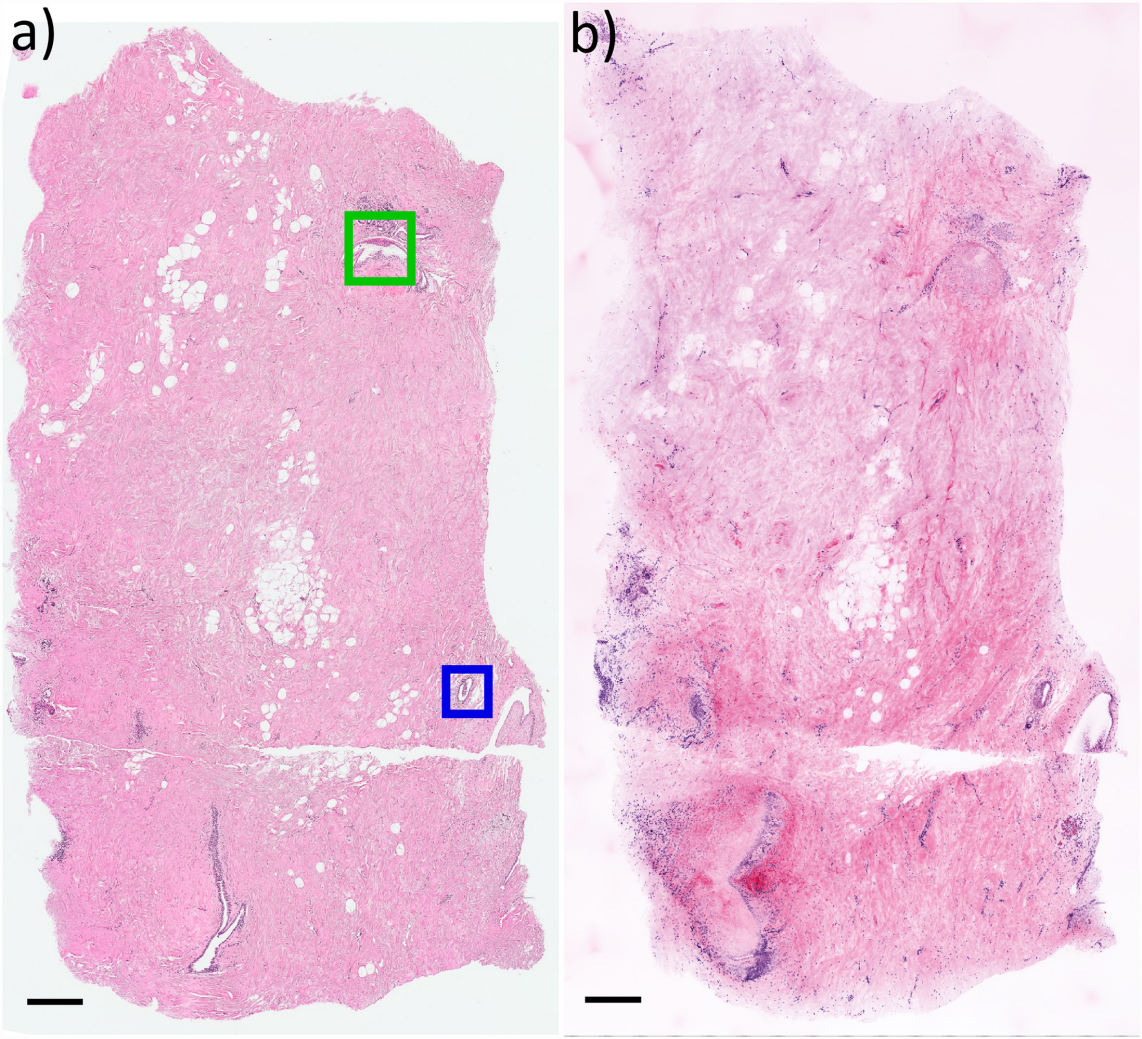
Comparison between histopathology and virtual transillumination H&E image generated by MPM. a) H&E histopathology image with apocrine metaplasia (green box) and benign breast duct (blue box). b) Corresponding virtual transillumination H&E image. The higher axial resolution of the MPM image better resolves individual collagen fibers as compared to the H&E section, an effect that could be reduced by using a lower NA objective. Due to minor tilting of the histological cutting plane, the left side of the H&E image is slightly deeper than the MPM plane and therefore transects more of the duct on the bottom left. Scale bar: 500 μm.

**Fig 3:**
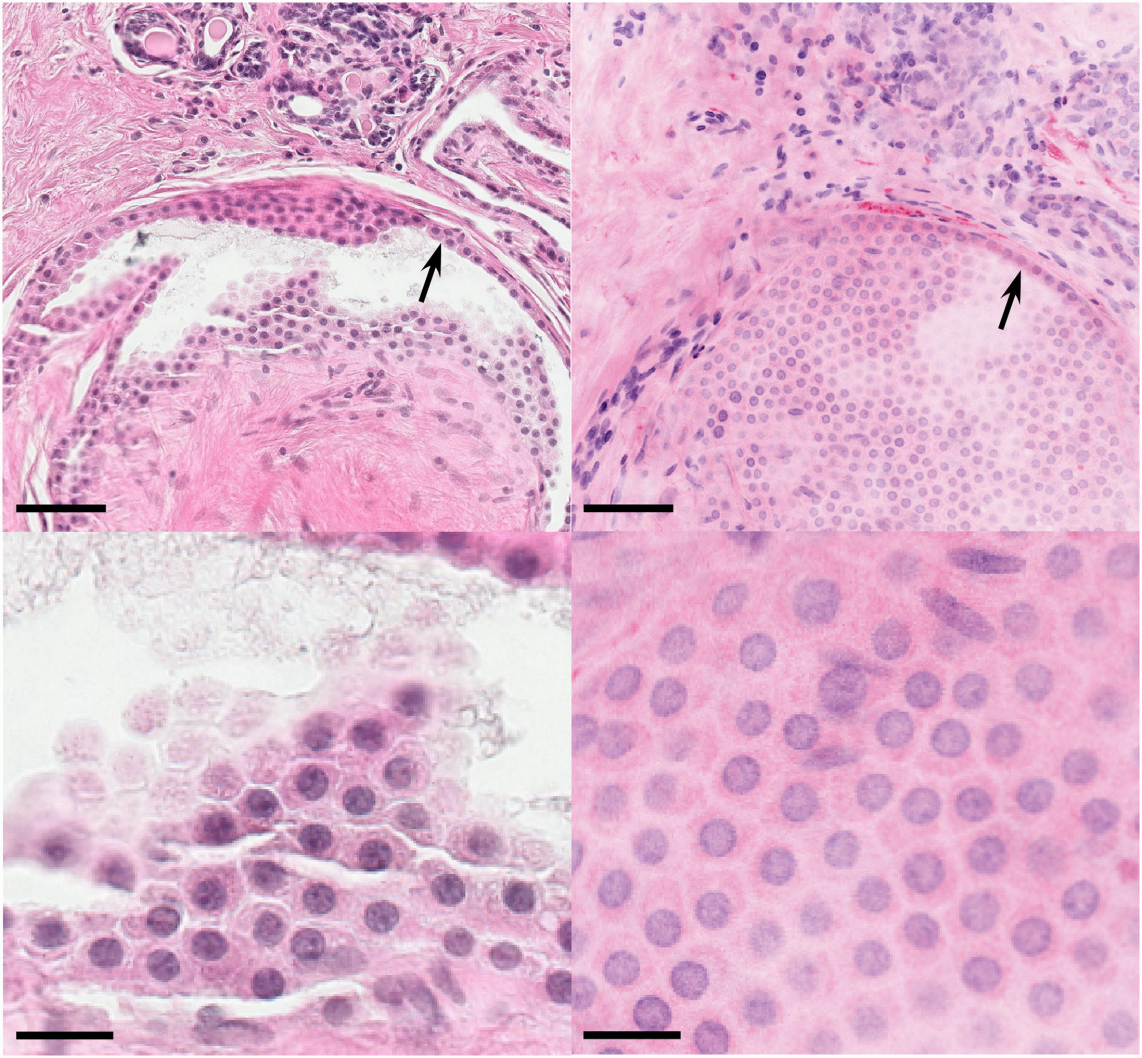
Enlargement of green boxed region in Fig 2 showing apocrine metaplasia on histopathology (left) and virtual transillumination MPM (right). In the low magnification view (top row), a distinct border (black arrows) separating metaplasia from normal breast tissue is apparent. In the enlarged view (bottom row), both modalities show large, uniform, round nuclei with vesicular chromatin and distinct nucleoli arranged in a thin sheet of hexagonally packed cells. Note that while MPM is non-destructive, the H&E sectioning has partially stripped away the metaplastic tissue and introduced crack artifacts (left). Scale bar: 75 μm (top), 20 μm (bottom).

**Fig 4:**
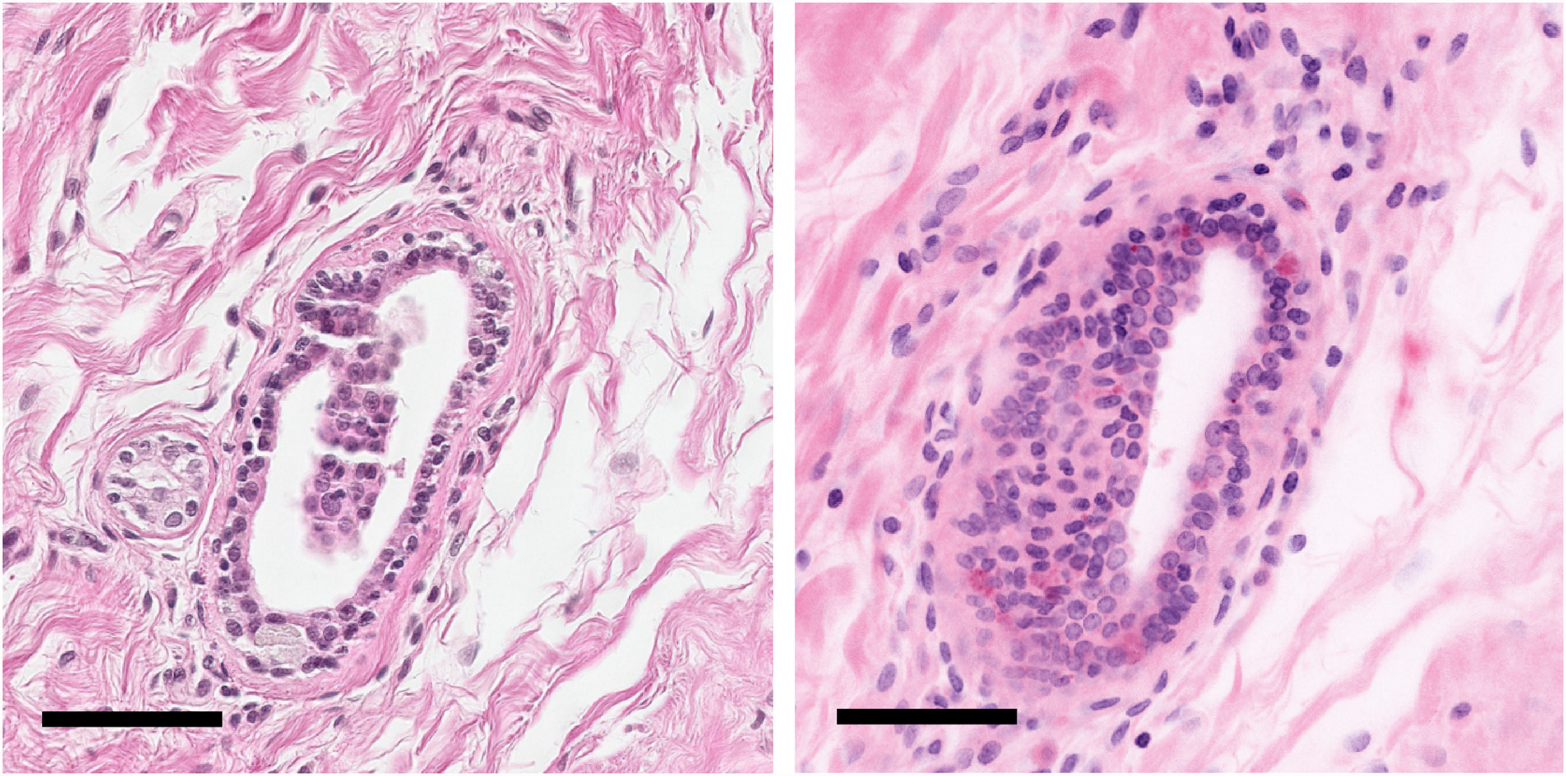
Enlargement of blue boxed region in Fig 2 showing a breast duct with some element of tangential sectioning. H&E histopathology (left). Virtual transillumination H&E image from MPM (right). Both modalities reproduce the duct structure as well as the surrounding collagen. Scale bar: 75 μm.

Fig 2 presents a comparison between the most superficial H&E section representing the entire specimen surface and the MPM Z depth plane of best agreement (in this case, approximately 10 μm below the first plane). Both depth planes are nearly identical, although in this example a minor tilt in the histological cutting plane causes the H&E image to be slightly deeper than the MPM image on the left side. We note that the significantly higher axial resolution of the MPM images results in a much thinner axial section than H&E, and correspondingly more distinct collagen fiber texture when viewed at lower magnification (Fig 2). The H&E histology was examined by an experienced breast pathologist. The specimen was found to contain several areas of benign pathology including prominent apocrine metaplasia (Fig 2 green box, and enlarged in Fig 3) and numerous breast ducts in both *en face* (Fig 2 blue box and enlarged in Fig 4) and transected (Fig 2., bottom left) views. The specimen is histologically benign, without *in situ* or invasive carcinoma.

### Comparison between virtual H&E methods

**Fig 5:**
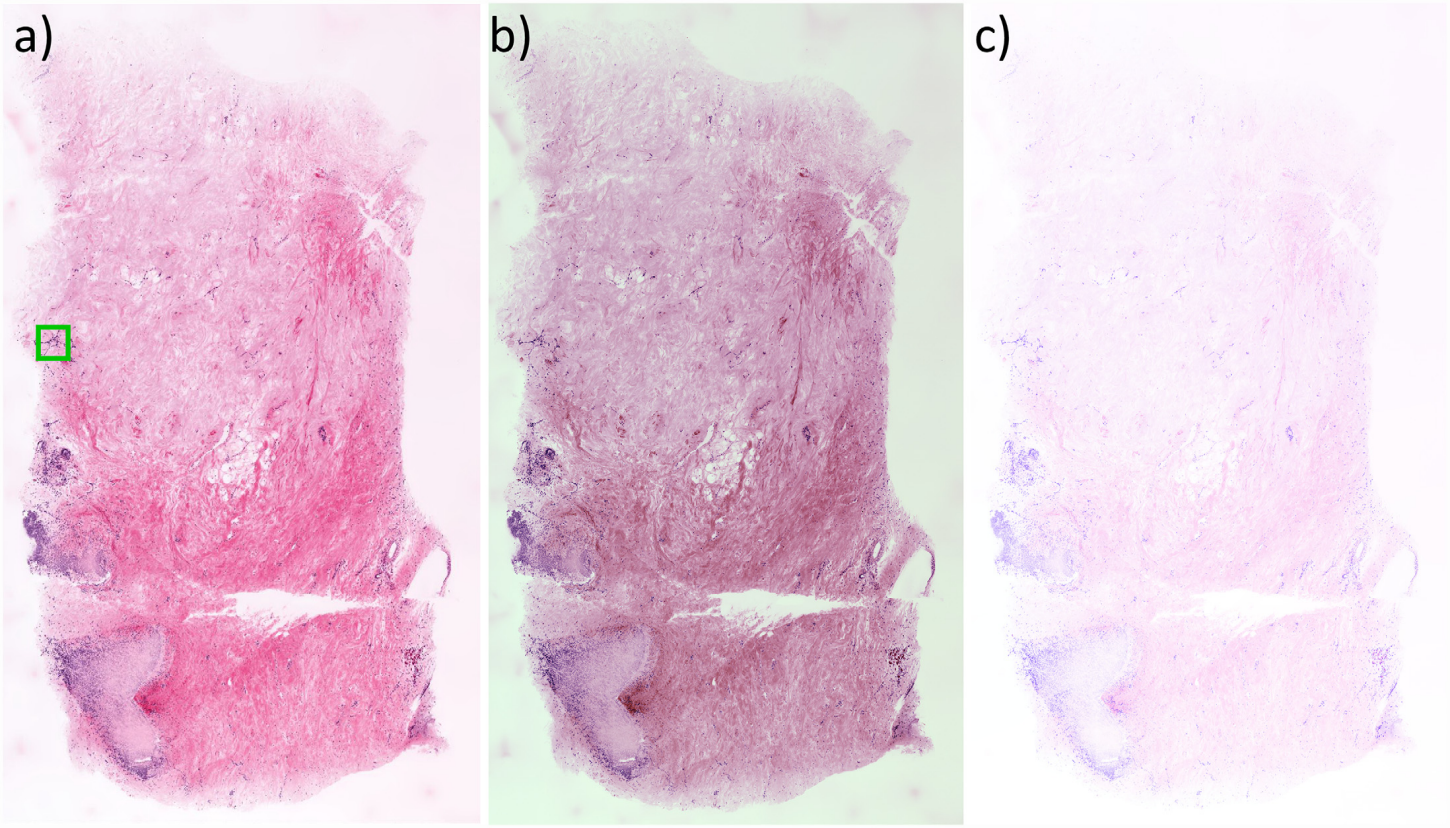
Virtual transillumination H&E images rendered from epi-fluorescence MPM imaging. a) Virtual transillumination Beer’s law algorithm. b) An additive method with nonlinear transfer function. c) Additive method with linear transfer function. The virtual transillumination algorithm enables both high contrast, realistic color rendering and perfect white point. In contrast, the additive method with nonlinear transfer function has reduced color accuracy due to the nonlinear transfer function shifting color slightly and minor green-shifting the backlight color to RGB [0.9 1.0 0.9], while the additive linear method has good color accuracy and perfect white point, but lower contrast. Of note, all three images span the entire 0.0 to 1.0 contrast range, and additional contrast enhancement is not possible without applying a nonlinear transfer function.

**Fig 6:**
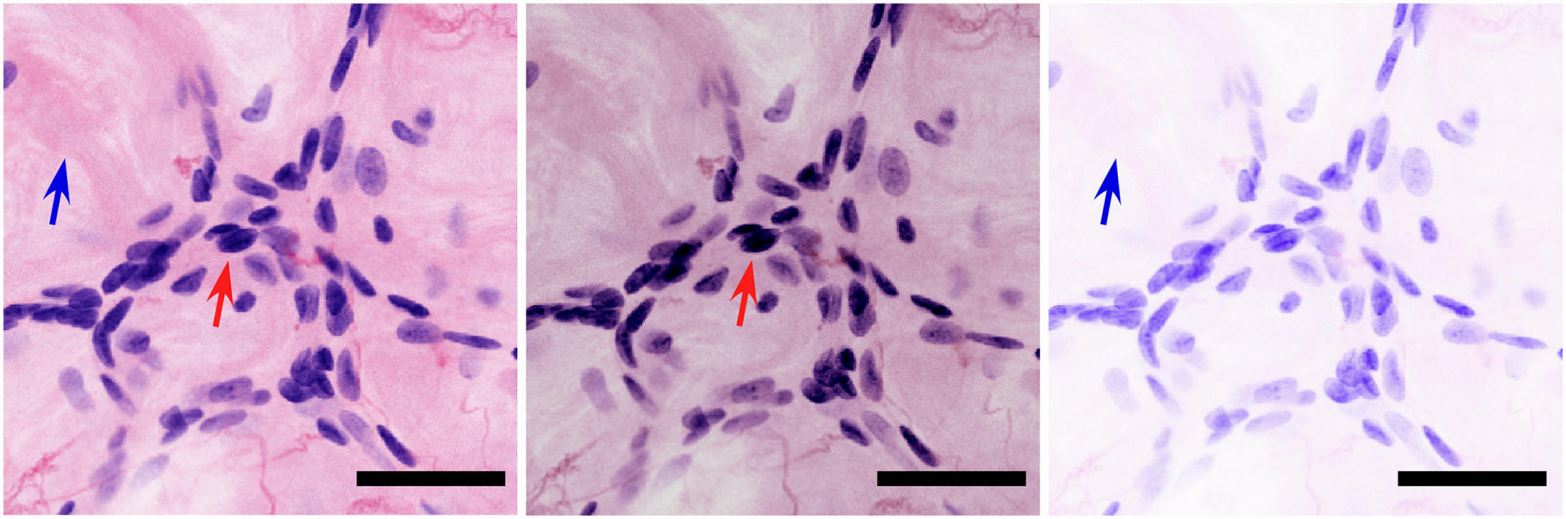
Enlargement of green boxed region in Fig 5 showing a cluster of darker staining nuclei. a) Virtual transillumination Beer’s law method. b) Additive method with nonlinear transfer function. c) Additive method with linear transfer function. Neither additive method has sufficient dynamic range to render both the nuclei (red arrow) and surrounding collagen fiber (blue arrow) accurately because of the strong spectral overlap between eosin and hematoxylin. Scale bar: 50 um.

The virtual transillumination algorithm enables a more physically realistic rendering of microscopy images as compared to additive methods. To evaluate these improvements, we selected an additional MPM image plane (nearest the approximate tissue surface) from the same specimen and processed it with an additive linear transfer function and an additive nonlinear transfer function as described in the Methods. The results are compared in Fig 5. These comparisons demonstrate the limitations of previous display methods, including inaccurate color and shifted white balance (additive method with nonlinear transfer function [9]) and reduced dynamic range (additive method with linear transfer function [12]). In contrast, the virtual transillumination method maintains an accurate white balance, accurate eosin and hematoxylin colors, and higher dynamic range than both additive methods. When viewed at high magnification (Fig 6), only the Beer’s law algorithm has sufficient dynamic range to render both the surrounding collagen and darkly staining nuclei without over or under saturation. The ability to visualize nuclear features is important for assessing neoplastic pathologies.

## Discussion

Transillumination white light microscopy is widely used for histological evaluation of human pathology. However, the need to transmit light through a sample requires physical sectioning into micron-thin slices through which light can pass with minimal scattering. The most widely used method to physically section tissue is fixation in formaldehyde, followed by paraffin infiltration and finally microtoming. Since this process is extremely lengthy (up to 1 day processing time), alternative methods such as cryosectioning (‘frozen sections’) are sometimes used when rapid (few tens of minutes) preparation and assessment is required. In the frozen section method, tissue is rapidly frozen at very low temperature and then microtomed. While frozen sections are faster in comparison to fixation, the freezing process introduces substantial image artifacts, and can be challenging with fatty tissues such as breast specimens that do not freeze easily [18–20].

A crucial advantage of molecularly specific imaging modalities such as multiphoton, confocal, structured illumination or Raman microscopy is the ability to reproduce established diagnostic features of classical histopathology, including the organization and composition of cell nuclei, without the lengthy delay associated with physical sectioning. By enabling visualization of established histological features, specimens can be evaluated by trained pathologists using accumulated experience in conventional histopathology. In this publication, we have demonstrated an algorithm for virtual transillumination microscopy, which combines optical measurements of tissue properties with computational light propagation in a GPU implementation to generate virtual transillumination images at video rates and with negligible frame latency. We have demonstrated the algorithm using multiphoton microscopy, however, this method should generalize to any molecularly specific imaging modality with sufficient axial sectioning. By avoiding the need to physically section tissue, specimens can be prepared more rapidly than even frozen section histology.

Previous studies have demonstrated H&E-like color mapping of fluorescence data to facilitate the interpretation of fluorescence images. However, aside from a recent patent [21], previously published methods have been based on the assumption that a transillumination microscope adds the transmission spectra of individual dyes together. Under this assumption, the image is generated by the sum of the colors of individual dyes at each pixel, which are assumed to be self-luminous with non-overlapping spectra. However, these assumptions are non-physical and are not a good approximation for dyes such as hematoxylin or eosin, which have substantial spectral overlap (both strongly absorb green light). As a result methods based on adding transmission spectra have reduced dynamic range and color fidelity because of the spectral overlap between eosin and hematoxylin. One possible solution is to implement a nonlinear intensity transfer function which improves the dynamic range of additive methods, but this degrades color fidelity and cannot fully reproduce the dynamic range of the Beer’s law method.

Instead of adding absorption spectra, the physical process of absorption in a transillumination microscope is described by Beer’s law: incoming light is exponentially attenuated according to dye concentration, rather than light being linearly emitted from the dye. Consequently, when multiple dyes are used in a sample, each applies a fractional multiplicative attenuation, ensuring that the sample cannot emit more light than it receives, nor transmit negative intensity. Furthermore, the virtual transillumination microscopy algorithm described here automatically reproduces the true exponentially-decaying-with-concentration intensity values seen in transillumination light microscopy without the need for any manual adjustment of sample parameters or post-processing, enabling true real-time operation. By incorporating Beer’s law into a computational light absorption model, we have shown that the dynamic range and color accuracy of virtual transillumination microscopy images can be substantially improved compared to previous rendering methods.

Finally, in order to facilitate wider investigation of virtual transillumination microscopy in scientific and medical applications, we provide an open source GPU implementation of the Beer’s law algorithm, as well as a simple example program and dataset that can be used to compare different rendering methods published in the literature. This code can be readily incorporated into existing microscopy software, enabling real-time virtual transillumination microscopy on existing fluorescence microscopes.
